# The Development of a Health Promotion Program for Unmarried Mothers Living in Residential Facilities Using Urban Forests: An Intervention Mapping Approach Based on the Transtheoretical Model

**DOI:** 10.3390/ijerph18168684

**Published:** 2021-08-17

**Authors:** Kyung-Sook Bang, Sungjae Kim, Gumhee Lee, Sinyoung Choi, Da-Ae Shin, Misook Kim

**Affiliations:** 1 Faculty of College of Nursing, The Research Institute of Nursing Science, Seoul National University, Seoul 03080, Korea; ksbang@snu.ac.kr (K.-S.B.); sung-jae@snu.ac.kr (S.K.); 2 College of Nursing, Seoul National University, Seoul 03080, Korea; csypass@snu.ac.kr (S.C.); olgar0108@snu.ac.kr (D.-A.S.); misook0218@snu.ac.kr (M.K.)

**Keywords:** unmarried mothers, residential facilities, forest program, transtheoretical model, program development, intervention mapping

## Abstract

Unmarried mothers living in residential facilities (UMLFs) in Korea face complex and challenging physical, psychological, and socioeconomic issues. This study developed a physical and mental health promotion program using urban forests for UMLFs based on the transtheoretical model and evidence. We utilized an intervention mapping approach (IMA) and assessed the needs of UMLFs by analyzing previous quantitative studies. Moreover, we conducted a qualitative hermeneutic phenomenological study involving nine participants. Based on the needs assessment, important and changeable determinants were identified; further, the program performance and change objectives were classified to achieve the program goals and establish the intervention strategy. We found that physical activity using forests, self-reflection using metaphors, five-sense activities, achievement activities using natural objects, building interpersonal relationships in the forest, and designing future plans, are desirable methods for improving the health of UMLFs. The IMA was deemed appropriate for the systematic development of health promotion programs for UMLFs through clear links among change objectives, theoretical methods, and practice strategies. These results should be applied to future intervention studies.

## 1. Introduction

The extramarital birth rate in most Organization for Economic Cooperation and Development (OECD) countries increased from less than 10% in 1970 to 24% and 41% in 1995 and 2018, respectively [1]. In addition, many low-income countries, such as in Latin America and sub-Saharan Africa reported rates of extramarital birth as high as 24% to 32% between 2014 and 2018 [2]. The increase in extramarital births is related to a social climate that facilitates cohabitation of various types of families without legal marriage procedures [1]. However, this family arrangement has also been linked to mothers’ lack of knowledge and financial constraints, especially in low-income countries [3]. Thus, the issues caused by extramarital birth are a global public health concerns and must be addressed urgently to avoid serious socioeconomic consequences [3,4]. In this study, mothers raising children without legal marriage are referred to as unmarried mothers (UMs) [5].

Compared with other OECD countries, the rate of UMs in South Korea (hereafter, Korea) in 2018 was 2.3% [6], whereas the number of UMs was 20,761 in 2019 [7]. The low rate of UMs in Korea is related to the societal view on family structure and pressures to adhere to conventional marriage customs [8,9], including prejudice and psychological resistance to UMs [9]. Thus, the socioeconomic status of UMs in Korea is not only unstable, but also threatens their childrearing and livelihoods, thus adversely affecting the health of UMs and their children [10,11]. Nevertheless, compared with previous trends in Korea, wherein over than 90% of UMs chose to place their children up for adoption, there has been a recent increase in the proportion of UMs who choose direct parenting. This is a relatively new phenomenon, representing a paradigm shift in Korean society [8].

These recent changes are related to the government’s support policy [10,12], which includes advocacy for maternity rights [12] and the expanded implementation of support policies for childrearing and self-reliance [8]. When UMs do not have a suitable place to raise their children due to poverty or lack of resources, the government and social agencies provide a place for them to raise their children [8,13]. These facilities are located nationwide, especially in metropolitan areas, and can be broadly divided into two types [13]. The first includes 22 basic living facilities (with a capacity of 561 persons) that support the safe delivery, recovery, and postpartum management of UMs during pregnancy and childbirth [13]. The second features 41 communal living facilities (with a capacity of 323 persons) that support raising and protecting infants under the age of 2 years [13]. Consequently, the number of residential facilities for UMs witnessed an 1.6-fold increase from 40 in 2007, to 63 in 2020 [13]. However, considering that 4.3% of UMs require residential facilities [7,13], there is a lack of residential facilities for UMs in Korea [10].

Although material support has increased, UMs living in residential facilities (UMLFs) face various challenges, similar to UMs worldwide [3,8]. UMLFs struggle with physical health care [14]; moreover, they are burdened with the responsibility of raising their children alone. Therefore, the prevalence of lifestyle-related diseases, such as the lack of sleep and an unbalanced diet, in UMs is relatively higher than that of the general population of the same age group [14]. In addition, despite their strong determination, many UMLFs experience depression and anxiety, as they assume the dual roles of breadwinning and childrearing without family and spousal support [15,16]. Thus, UMs in Korea experience multifaceted and multi-layered physical, psychological, and socioeconomic issues [10]. 

Nevertheless, prior studies highlighted that the support provided in residential facilities generally focuses on material assistance and does not comprehensively reflect the substantial needs of the UMLFs [10]. Moreover, the interventions provided for UMLFs thus far, have focused on the improvement of specific issues such as depression, anxiety [17], and parenting capacity [18]. These interventions have had limited effectiveness, as they have not been provided using a holistic perspective [17,18]. Additionally, prior studies reveal that UMLFs often surrender government-supported services because they are wary of other people’s views and fear exposing their identity [12]. Thus, interventions for UMLFs are not only highly inaccessible, but also lack a comprehensive and holistic perspective, that is based on the assessments of their needs [10,18]. Further, it must be noted that restrictions on external activities during the COVID-19 pandemic are likely to adversely affect the health of UMLFs [19]. Therefore, a program that comprehensively considers the environmental and circumstantial characteristics of today’s society as well as the special circumstances of UMLFs is required. 

Forests therapy is one of the most accessible methods, especially for people with low socioeconomic status, because the program utilizes currently existing and available urban forests [20]. Forests can promote positive changes through exposure to nature’s healing factors, such as air, sound, and green trees and healing ingredients such as terpene compounds [21,22]. Moreover, forests are effective in stimulating the mothers’ feelings of competence in parenting [21].

Evidence for the healing effect of forests has been investigated by previous studies [23,24,25,26,27,28,29]. Physically, activities in nature boost vitality and release energy [24,25]. Psychologically, the healing factors of the forest relieve negative emotions, such as anxiety, depression, and stress [24,26], while reducing negative cognition and providing opportunities for self-reflection [23]. Activities in forests improve social interaction by motivating individuals to express their thoughts and feelings [27,28]. Thus, as is evident from prior studies, programs using forests are effective in providing physical and psychological restoration. Moreover, such activities have a therapeutic effect even when using urban forests or indoor natural activities [23,26,29]. Therefore, interventions using the forest may be suitable for UMLFs, who require programs that apply a holistic perspective due to multidimensional issues.

Since a majority of the UMLFs issues are related to psychological difficulties [10,17], self-understanding based on in-depth self-reflection is a key factor in preparing programs for behavioral change in UMLFs [17]. The transtheoretical model (TTM) describes the process of psychological change in individuals with a focus on the cyclical nature of the participant’s cognition and behavior [30]. Moreover, it helps observe the internal changes in UMLFs. In contrast, an intervention mapping approach (IMA) [31] is an evidence-based intervention development model, which emphasizes the logical connection for each planned stage of intervention and establishment of theoretical ground. Further, it has multidimensional and dynamic characteristics that emphasize the development of a program that comprehensively considers individual, group, and environmental factors [31]; thus, this approach is useful to develop a program that considers the unique circumstances of UMLFs. The development of evidence-based programs for the health promotion of UMLFs is particularly necessary for community nurses or mental health nurses who provide nursing care for UMLFs, to ensure scientifically grounded nursing interventions. 

This study aimed to elucidate the process of the systematic development of a health promotion program for UMLFs, which not only integrates urban forests but is also based on the six-step protocol of IMA. In particular, this study focused on steps 1–5 of the IMA [31] and TTM [30] for the development of a program with high-level scientific grounding. Such a program aims to improve the physical and mental health of UMLFs who experience complex problems.

## 2. Methods

This program development follows the six steps of the IMA proposed by Bartholomew et al. [31]. The six steps comprise: needs assessment, preparing matrices of change objectives, selecting theory-informed intervention and practical strategies, program design and production, program implementation plan, and evaluation plan (Table 1). 

### 2.1. Step 1: Needs Assessment

#### 2.1.1. Conducting Basic Research and Review of Prior Studies 

In this study, to understand the stressors experienced by UMLFs and their responses comprehensively, complete studies conducted over the last 10 years were gathered to perform a systematic review [10]. Furthermore, the factors influencing parenting stress in UMLFs [32] and the composition and effects of interventions implemented to improve parenting competencies in UMLFs were reviewed in an integrated manner [18]. Moreover, through keywords such as unmarried mothers, mothers of adolescents, and pregnancy in teens, we collected literature using representative search engines in Korea, including the Korean Studies Information Service System (KISS), Korean Medical Database (KMbase), KoreaMed, National Discovery for Science Leaders (NDSL), and the Research Information Service System (RISS). We reviewed a study that discussed the intervention that contributed to the reduction in depression and anxiety in UMLFs [17].

#### 2.1.2. In-Depth Individual Interview 

The needs of UMLFs were analyzed using interpretive phenomenological analysis (IPA), based on the philosophy of hermeneutic phenomenology [33]. In-depth individual interviews were conducted with nine UMLFs to assess their needs. The interviewees included UMLFs who gave birth to and raised their own children; however, UMs who placed their children up for adoption, UMLFs who aborted their children, lived with their spouses or their families, and facility workers were excluded from the interview. The characteristics of the UMLFs who participated in the individual interviews for the needs assessment are presented in Table 2. The study was conducted with the approval of the institutional review board (IRB) of S National University (IRB number 2002/001-007). For recruitment of UMLFs, this study was introduced to the heads of the facilities where UMs resided through e-mail and telephone. Official promotional materials were then posted on the bulletin boards in the institutions, which enabled this study to be publicized. After assessing the promotional materials, UMLFs who wished to participate in the study contacted the researcher. Participants’ written consent was obtained after they received thorough explanations regarding the purpose of the study, participant anonymity, and confidentiality. The average duration of the interviews was 90 min. Participant needs were assessed based on a comprehensive evaluation of the practical difficulties experienced while raising children at the facility, the level of the UMLF’s awareness of the problem, and the UMLF’s coping abilities and resources. For a realistic and in-depth understanding of the needs of UMLFs, the interview guides comprised of questions such as: “what are the difficulties you face while raising your child?” “what do you do when you face difficulties?” and “what are your strengths and weaknesses as a mother raising your child?” Recorded interview content was transcribed, and meaningful words, phrases, and sentences were extracted, coded, and grouped into categories in the analysis process. The results of the analysis were checked using two UMLFs.

### 2.2. Step 2: Preparing Matrices of Change Objectives

In Step 2, the change objectives (COs) to be achieved through the program were determined based on the needs identified in Step 1. The COs of the program were structured into a matrix, which included determinants, performance objectives (POs), and their correlation with the COs. In the process of selecting determinants, we aimed to increase the scientific evidence and effectiveness of the program through a review of the factors affecting the health of UMLFs. Determinants were finally selected through iterative comparison and discussion of the health determinants and needs of UMLFs. Further, POs affecting the determinants were identified based on theoretical grounds.

### 2.3. Step 3: Selecting Theory-Informed Intervention and Practical Strategies

In Step 3, TTM was selected to identify changes in the participant. Based on the theory, COs were achieved through behavioral changes in UMLFs. Further, specific and practical intervention strategies were organized.

### 2.4. Step 4: Program Design and Production

In Step 4, the content and order of the overall program were organized based on the specific COs and practical intervention strategies determined in the previous steps. The theme of each session was determined by considering the POs and the TTM that would reflect the participant’s changes. For the program implementation, urban forests, natural objects, and materials used in the activities were selected after considering the accessibility of the UMLFs. 

### 2.5. Step 5: Program Implementation Plan

In Step 5, the program’s implementation was determined by assessing the feasibility of the implementation. In this study, the specific implementation method of the program was systematically reviewed by experts in related fields. To increase the level of content validity evidence, this study recruited three experts with high levels of scientific and applied knowledge and experience on the construct of interest. Furthermore, two experts with experience in test construction and psychometrics evaluated the items using a different perspective than that of the recruited content experts [34]. Thus, in this study, experts in related fields, i.e., those that possessed an in-depth understanding of UMLFs and experience in forest healing interventions, were selected to evaluate content validity.

### 2.6. Step 6: Evaluation Plan

In the final step, an evaluation plan for behavioral changes that may occur in UMLFs was developed. In this step, the process of whether the program was adequately applied and implemented among UMLFs is evaluated, and a study design and measurement parameters are planned to evaluate the effectiveness of the program.

## 3. Results

### 3.1. Step 1: Needs Assessment

#### 3.1.1. Analysis of Prior Research

Most UMLFs experience threats to their physical health owing to chronic fatigue from raising their children alone, without the support of their spouses or family [10]. In particular, UMLFs experience depression and anxiety, owing to a sense of a lost existing social status and the burden of raising their children independently [10,17]. They experience low self-esteem due to social criticism and the stigma of giving birth to and raising children without being married in Korean society, which has a deep-seated patriarchal culture [10]. Additionally, most UMLFs experience chronic parenting stress, owing to the burden of breadwinning and adapting to maternal roles, since they cannot expect help from their family or the father of their child [10,32]. Therefore, prior studies reveal that UMLFs experience various psychological difficulties in addition to the threat to physical health [10,32]. Nevertheless, due to the lack of psychological support [10], interventions to enhance a sense of psychological stability are warranted. Furthermore, our results emphasize the need for introspection and emotional expression to control depression and anxiety in UMLFs [17], as well as interventions to assess and improve physical, mental, and social health conditions to relieve parenting stress [32].

#### 3.1.2. In-Depth Individual Interview

The average age of UMLFs was 28.7 (20–37) years, and the average age of their children was 19.3 (3–36) months. The UMLFs reported feeling depressed, sad, and guilty due to their experiences of raising children in a facility, without spousal or family support. Even if they were dissatisfied with facility workers or other UMLFs, they were more concerned about the disadvantages of living outside the facility and being psychologically withdrawn because they were unable to endure their living conditions. In addition, UMLFs reported feeling anxious, as they were not able to trust others due to their painful life experiences; they were afraid and angry about their uncertain future and criticism from others. The UMLFs reported that they felt guilty about not being able to provide enough for their children due to their current situation. However, they reported feeling anger when their children asked them for something. Through in-depth individual interviews, it was confirmed that the UMLFs were living with chronic mental health issues such as depression, anxiety, and low self-esteem, which contributed to their parenting stress. Through a needs assessment, we found that UMLFs required pain relief as well as an object of psychological consolation.

#### 3.1.3. Establish Program Outcomes

Specific outcome variables to evaluate changes in UMLFs were selected based on the priorities identified in the review of previous studies and the qualitative data analysis. Consequently, physical health, depression, anxiety, self-esteem, and parenting stress variables were selected.

### 3.2. Step 2: Preparing Matrices of Change Objectives

The ultimate goal of this program is an improvement in the quality of life of the UMLFs. The specific objective of the program is a statistically significant reduction in the physical and mental health problems of UMLFs at the end of the program, compared with their state prior to program participation. This program aims to objectively assess the health status of UMLFs and confirm the effectiveness of the intervention program by scientifically measuring their improvement in health status after the intervention.

#### 3.2.1. Determinants

Based on the analysis of previous studies and in-depth individual interviews on personal determinants affecting the health status of UMLFs, knowledge and skills affecting the relief of distress in their lives, and improvement of health status, helplessness, and the attitude of resignation to the reality that they have no one to rely on were selected. For environmental determinants, accessibility to available resources according to socioeconomic status was selected (Table 3). 

#### 3.2.2. Performance Objectives

The specific POs for improving the mental and physical health status of UMLFs were developed after considering the characteristics of the determinants and outcome variables. The details of the six POs are as follows: PO1: determination of improvement in mental and physical health using forests; PO2: using forests for self-reflection of one’s inner being; PO3: attempts at self-care using forests; PO4; experiencing emotional comfort in forests; PO5: practicing positive child rearing based on cooperation and communication learned in the forests; and PO6: designing and planning a positive future with a child using forests.

In particular, PO1 and PO2 focus on reducing anxiety (present), PO3 on the enhancement of self-esteem, PO4 on depression, PO5 on parenting stress, and PO6 on anxiety (future) reduction. The order of these POs was based on a report that revealed that individual changes through the use of forests occur in the order of body, emotion, cognition, and behavior [21], and a study that structured the hierarchy between outcome variables [10]. In other words, the therapeutic functions of the forest are expected to have an overall positive effect on physical health and negative emotions such as depression and anxiety, self-esteem, and stress [26,35]. However, we expected that the reduction in anxiety, a variable related to emotions, would occur before the improvement of self-esteem, a cognition-related variable; this was reflected in the organization of the sequence of each session [10]. Thus, the composition of POs and COs was systematically developed, considering the therapeutic characteristics of the forest and the characteristics of the outcome variables. The COs for implementing the POs of this program were constructed through in-depth discussions based on a literature review. The healing effects and the application method of forests according to individual variables are discussed in detail in Step 4. Table 3 shows the metrics for the effects and methods.

### 3.3. Step 3: Theory-Based Methods and Practical Strategies

#### 3.3.1. Related Theory

Knowledge, skills, attitudes, and accessibility are important determinants in the metrics of this study. The TTM proposed by Prochaska and DiClemente et al. [30] was employed in this study to induce and observe changes in UMLFs for the future’s intervention study. The TTM proposes five steps of precontemplation, contemplation, preparation, action, and maintenance, and 10 theoretical methods, which influence the participant’s cognition, emotion, and evaluation to induce positive changes [30]. In this study, it was deemed appropriate for the composition of strategies for change among the participating UMLFs (Table 4).

#### 3.3.2. Practical Strategies

Since the intervention strategy provides information to improve the knowledge of UMLFs, it is suitable for enhancing their level of understanding. Further, small group lectures with audio-visual materials were utilized [36]; moreover, demonstration, practice, and supervision were employed as major strategies to improve skills [36]. Group discussion, support, and motivation, which are suitable to promote internal motivation, were used as strategies for attitudinal changes [36]. Group discussion and social support were used as strategies to increase accessibility and availability (Table 4).

### 3.4. Step 4: Program Design and Production

To achieve COs for the health promotion of UMLFs, a program with eight sessions was designed, and themes that fit the COs for each session were selected. Moreover, strategies and tools that can effectively deliver the program were selected and organized for each session (Table 5).

#### 3.4.1. Generate Program Themes, Components, Scope, and Sequence

The eight-session health promotion program using urban forests was implemented for eight weeks, with one 90 min-session per week. The sequence of sessions followed the five-change steps of the TTM [30]. In the precontemplation step, UMLFs determine their changes and decide to participate in the program. In the next step, contemplation and preparation, UMLFs have expectations and hopes for change, and are motivated. This step corresponds to sessions 1 and 2 of the program, and the themes of this step are pleasant meeting in forests and forest of reflection. In the next step, UMLFs acquire new healthy behaviors, correct problem behaviors, and continue to change their healthy behavior. Sessions 3–7 correspond to this step with themes forest of care, forest of achievement, forest of comfort 1, 2, and forest of happiness. In the final step of maintenance, UMLFs continue to change their behavior and develop hopes for a healthy future. This step corresponds to session 8 and concludes the program with the theme of forest of hope. (Table 5).

#### 3.4.2. Select or Design Practical Application to Deliver Change Methods

The specific contents of individual sessions were designed by considering the POs and COs based on a thorough literature review.

##### Methods of Physical Health Promotion

This program aimed to promote the physical health of UMLFs by including physical activities in the sessions. In particular, session 1 was designed to develop an interest in the UMLFs to select forests as a tool for restoration, provide instructions on specific methods of using the forests for therapeutic purposes, and improve their knowledge level and motivation for participation based on the proposition of TTM [30]. Specific activities to improve physical health include resting in the forest, walks, barefoot walking, and foot baths (Table 5) [26].

##### Methods of Reducing Anxiety through Emotional Relaxation

Sessions 1 and 2 were designed using forests and natural objects to allow UMLFs to experience emotional relaxation and anxiety reduction through self-reflection and expression of emotions [17]. Specific activities included finding a tree that resembles oneself and talking to the tree about emotions, drawing one’s face using natural objects, and sharing one’s emotions (Table 5) [37]. 

##### Methods of Improving Self-Esteem through Self-Reinforcement

In sessions 3 and 4, UMLFs focused on self-care while experiencing the forest through meditation in the forest and walking using the five senses [21,26]. Participants experience a sense of achievement through creating horticultural crafts using natural objects; further, their self-esteem can be improved by giving and receiving compliments to and from other members (Table 5) [21,26]. 

##### Methods of Reducing Depression through Emotional Stability

In sessions 5 and 6, UMLFs are encouraged to modify their negative emotions through five-sense activities in the forest (e.g., walking, and aroma hand massage) and experiencing psychological comfort by walk in an urban forest path and share the experience of taking pictures in the forest [21,26,37]. In the online method, through forest meditation and walking through urban forests, physical activity will be encouraged to recharge as well as reduce depression of UMLFs based on prior studies [26]. Furthermore, the program was designed for UMLFs to practice mutual self-care, independently (Table 5).

##### Methods of Reducing Parenting Stress through Understanding and Acceptance of the Importance of Life

In sessions 7 and 8, the experiences of parenting stress are shared; the UMLFs practice effective communication and exchange parenting methods to reduce parenting stress [10]. Additionally, UMLFs are encouraged to set specific goals for the future by focusing on areas that they feel uncertain and insecure about (Table 5) [37]. 

#### 3.4.3. Prepare Plans for Program Materials

The urban forest, which is the physical space for the implementation of the program, is selected in a place accessible to the UMLFs. If program activities cannot be conducted in urban forests (e.g., during rainy weather or infectious disease pandemics), the online medium will be adopted. In the case of the online program, the items required for each session are delivered to the facility where the UMLFs reside, and guidance is provided for the preparation of these items prior to the session. While conducting the program online, a video conferencing application that can be accessed through web browser as well as mobile is used; further, the UMLFs are notified on this application prior to the commencement of the program to facilitate their access and use.

### 3.5. Step 5: Program Implementation Plan

#### 3.5.1. Identify Potential Program Users

The beneficiaries of this program are UMLFs who are raising children. This program is intended for adult female UMLFs who can cope with common risks and workers in collective housing facilities. UMLFs’ families of origin or fathers of children are not eligible for the program. Among UMLFs, those experiencing severe depression or anxiety, who are judged to require acute hospitalization and drug treatment are not prioritized for participation in this program.

#### 3.5.2. Staff Training

The staff were trained in accordance with the National Institutes of Health Behavior Change Consortium recommendations [38]. Delivering standardized programs requires education and training to prepare the general staff, who have varying educational levels, skills, and professional backgrounds. In this study, the quality of education was ensured by two professional leaders who supervised four individual staff members, who shared educational materials through regular meetings, and observed the implementation of interventions through a pilot program (role play). In addition, all staff members participated in workshops and related academic conferences. Meanwhile, the program requires at least two experts consisting of the main and assistant hosts; moreover, they should complement each other to enhance the completion of the program implementation. Thus, program operators should consider the environment in which the program is to be delivered by surveying the urban forest in which the program will be provided, and determine whether the program can be operated. 

#### 3.5.3. Program Validation

In this study, the evaluation of the program’s content validity index (CVI) was assessed by five experts comprising two professors in psychological nursing, one professor in pediatric nursing, one professor in forestry, and one professor in community health nursing, who had research experience in forest healing. The CVI consists of eight questions and is measured using a 4-point Likert scale from 1 = not valid at all, to 4 = very valid. The contents of the CVI were constructed based on items such as participant characteristics, the feasibility of implementation, possibility of online/offline application, number of sessions and sequence of composition, theoretical framework, forest healing factors, and the possibility of change in participants. Item-level content validity index (I-CVI) is the evaluation of each item by experts; points 1 and 2 are scored as 0 and points 3 and 4 are scored as 1; further, the expert’s I-CVI for this program ranged from 0.8 to 1.0. The scale’s content validity averaging method (S-CVI/Ave), which is the average value of I-CVI, was also applied. It is considered valid if the value is 0.9, or higher [39]; as the S-CVI/Ave for this study was 0.93, the content validity of the program was confirmed. Furthermore, following the opinions of experts, the self-praise and encouragement in session 4 were modified to give self-praise and praise to members for their horticultural craft products to promote social relationships among members.

### 3.6. Step 6: Evaluation Plan

The final step was to develop an evaluation plan. The evaluation plan was prepared for the future study, and it is conducted by separating the process evaluation and the effect evaluation.

#### 3.6.1. Planning Designs for Process Evaluation

The key factors of the process evaluation are context, reach, dose, fidelity, implementation, and recruitment [40]. In context, evaluation is performed through the program or message that is provided to UMLFs; in reach, the extent to which the UMLFs reached the intended objectives is evaluated. In dose, the frequency of delivery of the program, occurrence of any deletion or irregularity, and number of UMLFs present is evaluated. Regarding fidelity, the program is evaluated to assess if it was delivered according to the goals and objectives. In implementation, the program’s contribution and acceptance by UMLFs will be evaluated; in recruitment, the approach uses to engage program participants will be evaluated. Moreover, at the end of every session, a satisfaction survey of each session is conducted with the UMLFs. If the program was run online, the course evaluation for the online program is conducted separately [41].

#### 3.6.2. Planning Designs for Effect Evaluation

##### Study Design

To evaluate the quantitative effectiveness of the program, the study will be conducted with a non-equivalent control group pretest-posttest design after considering the feasibility of the study, as residential facilities are scattered across Korea and the number of residents per facility (approximately 10) is small [7,13]. Quantitative analytical methods for analyzing intervention effects have limitations in terms of observing changes resulting from intervention participation [42]. Therefore, to observe the changes in UMLFs, using a mixed study method in conjunction with a qualitative study method is desirable. The participants of this study are UMLFs to secure homogeneity between experimental and control groups. Moreover, participants of this study did not previously participate in activities related to mental and physical health promotion using urban forests, neither did they participate in general programs provided at residential facilities.

##### Participant’s Calculation and Data Analysis

The G*power 3.1 program was used to determine the number of participants required for the study. As the t-test and repeated measures analysis of variance (RM ANOVA) are essentially the same [43], the minimum number of participants was set at 17 per group. The effect size of forest therapy on human negative emotions was reported to be medium or high [44]. Therefore, the significance level was set at 0.05, the effect size at 0.25 (median), the power at 0.80, the correlation coefficient at 0.5, and the number of repetitions at 2. Considering the dropout and incomplete response rate of 20% [45], the sample size was finally set to 21 participants per group, resulting in 42 participants. A pre-test will be conducted before commencing session 1 of the program; further, a post-test will be conducted after completing session 8 of the program. The statistical package for the social sciences (SPSS) for Windows version 23.0 is used for all data analyses, and statistical significance was set at *p* = 0.05. The qualitative data were analyzed using qualitative content analysis [46]. Descriptive statistics, ANOVA, and RM ANOVA can be applied to quantitative effect evaluation in further studies. 

##### Measurement

The questionnaire is administered before and after the intervention. It comprises 84 questions pertaining to physical health, depression, anxiety, self-esteem, parenting stress, and general characteristics. Participants are required 20–30 min to complete the questionnaire. Specifically, subjective health status is measured using one item, physical health is measured using a 14-item physical health scale (PHS) (Cronbach’s α = 0.74~0.87) [47], and depression is evaluated using a 12-item Korean screening tool for depressive disorders (K-DEP) (Cronbach’s α = 0.95) [48]. In addition, anxiety, self-esteem and parenting stress are measured using the 11-item Korean screening tool for anxiety disorders (K-ANX) (Cronbach’s α = 0.96) [48], the 10-item Rosenberg self-esteem scale (SES) (Cronbach’s α = 0.75~0.87) [49], and the 36-item Korean version of the parenting stress test-abbreviated (K-PSI-4-SF) (Cronbach’s α = 0.93) [50], respectively. 

##### Ethical Considerations

Since UMLFs are a vulnerable group, the content and method of this study must be approved by the Ethics Review Committee. Participants are provided detailed explanations of the purpose of the research, the process of programs, the anonymous handling of the written consent, confidentiality, voluntary participation, and possibility to withdraw from the study at any time without any disadvantage. They are reassured that in the process of completing the questionnaire, unnecessary personal information such as name and address will not be investigated, and the collected data will be statistically analyzed with a unique serial number. To prevent leakage of personal information, the collected data is stored in the form of a file with an access password, and is not viewed by anyone other than the research director.

## 4. Discussion

In this study, an eight-session health promotion program using urban forests was developed for UMLFs. Hermeneutic phenomenology was used to assess the needs of UMLFs, who experience complex and diverse psychological problems. In particular, IPA was selected as an analysis method to reflect the philosophy of hermeneutic phenomenology. IPA is an interpretive and exploratory analysis method that enables sophisticated and nuanced analysis with its core concern being psychological [33]. Therefore, it is judged as suitable for an in-depth evaluation of the needs of UMLFs who face various mental health issues. This is also supported by the argument that IPA, as an analysis method, is appropriate for groups of people with highly emotional and complex psychological needs, who can be considered as the vulnerable stratum [51]. Moreover, only nine UMLFs participated in the qualitative analysis to assess the needs of UMLFs. The hermeneutic phenomenology intensively explores participants’ experiences and lifeworld [42]. Moreover, it considers less than 10 participants as appropriate for a study [42]. Based on the philosophy of hermeneutic phenomenology, this study collected and evaluated their needs until data saturation was reached; thus, the analysis of nine participants was sufficient to assess UMLFs’ needs.

The distinctive strength of the program developed in this study was that it was based on the TTM, a theory suitable for establishing and evaluating changes in UMLFs. The strength of the TTM is that it provides knowledge on behavioral changes by promoting gradual changes and underlying motivating factors [30]; hence, the model is suitable for examining the motivation for change in UMLFs as well as observing behavioral changes by applying strategies of behavioral changes to their health. In this program, the process of attaining motivation, learning, acquiring activities for change, and maintaining healthy behavioral changes has been systematically developed to enable setting detailed and specific targets for each session and observing the UMLFs’ achievement of targets. For example, in sessions 1 and 2 of the program, consciousness-raising (obtaining the facts) or self-reevaluation were applied, which are appropriate change strategies for the precontemplation and contemplation stages. In sessions 5 and 6, social liberation or helping relationships were applied, which are appropriate change strategies for the action stage. Thus, the detailed design enabled gradual changes by considering motivations and steps of change for the participating UMs. Additionally, since the variables that effect change are gradually maximized according to the program provision, and the therapeutic characteristics of the forest were considered in the program [10,21], we hope that the intervention time can be shortened and the resources required can be reduced in the future.

Another strength of this study is that as an intervention development method for behavioral changes, the IMA was applied, which identifies the needs of the participants. Moreover, COs and intervention strategies were selected based on the identified needs [31]. Thus, the proposed program is different from other programs as it is developed according to the stages of IMA; moreover, the sessions of interventions were designed according to the objectives of behavioral change and flow in the TTM stage [52]. Moreover, since an integrative program was designed with the combined application of the TTM and IMA, a high level of program effects can be expected; the program also has a firm scientific basis for application and evaluation.

Furthermore, this study is meaningful as it has a high possibility of implementation; moreover, it was developed by evaluating the content validity of the program, with the assistance of experts, to adopt activities that are practically applicable to UMLFs. In particular, to assess the needs of UMLFs [31], the first stage of IMA, previous studies and data from in-depth interviews with UMLFs were used. Hence, since the needs of UMLFs were identified with sensitivity and the program was prepared based on the identified needs, its contents reflect the real needs of UMLFs. In other words, the application validity of this program was ensured because it was based on a comprehensive assessment of UMLF needs [10,17].

This program is highly applicable as it enables both online and offline applications in cases where face-to-face intervention are not quite possible, such as the COVID-19 pandemic. In particular, for UMLFs living in groups, face-to-face intervention has a potential risk of exposure to infection; thus, an online program version eliminates this risk. Based on studies that reveal the association between an increase in depression and the restrictions on external activities to suppress the spread of infectious diseases [19] as well as the need for appropriate interactions and activities for health promotion [20,35], the findings of this study have significant implications for the current situation. In particular, the online program allows UMLFs to autonomously experience nearby urban forests through assignments that help them learn to use urban forests, thus leading to positive effects when coping with stressful situations. The grounds for these discussions can be found in previous reports [18,32] through changes in the participants and development and reinforcement of internal capabilities through which UMLFs are equipped to tackle problems independently and increase their understanding of available resources.

The limitation of this study is that the effectiveness of the proposed intervention program was not evaluated after program development. Moreover, for the feasibility of the study, a small number of nonequivalence pre- post-experimental designs were proposed to evaluate the effect. This is a limitation as the design of an experimental study must be scientific, while considering the possibility of approach and implementation. In addition, the challenge of selecting and training a mediator may also be a limitation of this study.

However, this study is significant as the program was developed through the application of a comprehensive needs-assessment of UMLFs; moreover, it was based on the theory of behavioral change and adhered to a systematic intervention development method. Additionally, since the program developed in this study can be applied using both offline and online mediums, the program considers the needs and demands of today’s circumstances, in which external activities are restricted [19,41]. This program was developed with strict adherence to IMA based on the TTM. It required understanding of the situation of UMLFs and conviction in human behavior changes; thus, community nurses or mental health nurses who possess this knowledge should develop an interest in UMLFs. Since UMLFs are a vulnerable group, considering the ethical aspects of interventions involving them is crucial. This study therefore comprehensively considers the ethical aspects in the preparation of interventions applicable to UMLFs. The results of this study are expected to be useful to a variety of professionals working with UMLFs in their communities; moreover, this study will help generate a body of evidence-based knowledge applicable to UMLFs.

## 5. Conclusions

In this study, the steps and principles suggested by IMA and TTM were systematically structured with forest healing factors to develop a health promotion program using urban forests for UMLFs. This scientifically designed program was developed based on the various needs of UMLFs with a comprehensive assessment of their mental and physical health status; it is therefore expected to contribute to the health promotion of UMLFs from a holistic perspective. Based on the results of this study, for the health promotion of UMLFs, strategies such as physical exercise in the forest, self-reflection using metaphors, five-sense activities, achievement activities using natural objects, building interpersonal relationships in the forest, and designing future plans are desirable. Regarding further research, we propose an intervention study based on the results derived from this study.

## Figures and Tables

**Table 1:** Steps of the intervention mapping framework.

	Step	Tasks
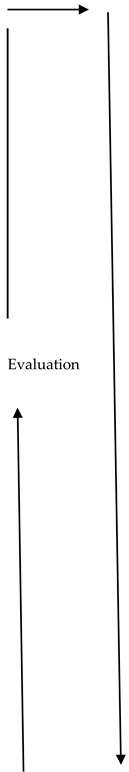	Step 1 Needs Assessment	Establish a participatory planning group; Conduct the needs assessment and problem analysis; Assess health and environment conditions; Establish program outcomes.
	Step 2 Preparing Matrices of Change Objectives	State expected changes in health status; Specify the determinants; Specify the performance objectives; Create matrices of change objectives.
	Step 3 Theory-Based Methods and Practical Strategies	Identify a related theory; Identify practical strategies based on the theory.
	Step 4 Program Design and Production	Brainstorm available materials; Generate program themes, components, scope, and sequence; Prepare design document and protocol; Develop program materials.
	Step 5 Program Implementation Plan	Identify potential program adopters and implementers; Specify adaptation, implementation, and sustainability of performance objectives; Evaluate adaptation, implementation, and sustainability of performance objectives; Confirm methods and strategies.
	Step 6 Evaluation Plan	Design intervention for adaptation and implementation; Develop an evaluation model; Develop process and effect evaluation questions; Complete the evaluation plan.
Implementation		

**Table 2:** General characteristics of participants.

No.	Age of Mother (Years)	Education Level	Job Status	Length of Residence in Facility (Months)	Age of Children (Months)	Sex of Child
**1**	24	College dropout	Unemployed	34	17	Girl
**2**	32	High school	Employed	36	36	Girl
**3**	28	Attending college	Unemployed	20	15	Girl
**4**	35	High school	Unemployed	28	18	Boy
**5**	23	High school	Unemployed	36	3	Girl
**6**	34	High school	Unemployed	13	15	Boy
**7**	20	High school	Unemployed	7	7	Boy
**8**	25	High school	Unemployed	21	34	Boy
**9**	37	College	Unemployed	24	29	Girl
**Mean**	28.7			24.3	19.3	

**Table 3:** Matrix of the Urban Forest Program for Unmarried Mothers.

Performance Objectives, Unmarried Mother will:	Target Variables	Personal Determinants			Environmental Determinants
		Knowledge	Skill	Attitude	Availability and Accessibility
PO1. Determine the improvement of their mental and physical health using forests	Anxiety	CO1.1. Understand the therapeutic functions of the forest and the method of using forests.	CO1.2. Express methods of using forest healing factors.	CO1.3. In terms of mental and physical health promotion, the contribution of forests is examined.	CO1.4. Identify the surrounding forest environment available for use.
PO2. Use forests for self-reflection of one’s inner being	Anxiety	CO2.1.a. Understand the positive role of forests in relieving anxiety and stress. CO2.1.b. Understand that the metaphor of forests can be used for non-threatening introspection.	CO2.2.a. Use forest healing factors to reduce anxiety. CO2.2.b. Express oneself safely using the forest metaphor.	CO 2.3.a. Develop the expectation of using forests to alleviate anxiety CO 2.3.b. Develop the expectation of using forests to express oneself safely.	CO2.4. Make continuous attempts to connect with nature.
PO3. Make attempts of self-care using forests	Self-esteem	CO3.1.a. Re-evaluate oneself through the forest. CO3.2.b. Understand the need for self-care.	CO3.2.a. Practice self-care through five-sense activities in the forest. CO3.2.b. Make attempts of self-encouragement and achievement using natural objects.	CO3.3. Convince oneself that self-reinforcement and a sense of accomplishment can be acquired through green activities.	CO3.4. Develop a plan for green activities that can be conducted indoors.
PO4. Experience emotional comfort through forests	Depression	CO4.1.a. Learn the method of emotional comfort using forests. CO4.1.b. Understand the need to care for oneself and others.	CO4.2.a. Experience sufficient relaxation and psychological comfort using five-sense activities in the forest. CO4.2.b. Practice caring for oneself and others.	CO4.3.a Be convinced that the use of forests can provide introspection and psychological comfort. CO4.3.b. Confirm that one can practice mutual self-care with peers using the forest.	CO 4.4. Identify five-sense activities that one can practice independently.
PO5. Practice positive child rearing based on cooperation and communication learned in the forest	Parenting Stress	CO5.1.a. Recognize the value of raising children using a forest metaphor. CO5.1.b. Prepare a plan to collaborate with peers in raising children through brainstorming.	CO 5.2.a. Use the forest to express the child’s value and preciousness. CO5.2.b. Express positive parenting strategies in a socially favorable way of communication.	CO 5.3.a. Experience the sense of reward in life with children. CO 5.3.b. Improvement in mutual support and coping capabilities can be expected in child rearing through increased understanding, communication, and cooperation among peers.	CO 5.4. Proactively and persistently apply the measures to reduce parenting stress.
PO6. Design and plan a positive future with the child using forests	Future Anxiety	CO6.1. Use the forest to identify good parenting plans.	CO6.2. Demonstrate a blueprint for a positive future with the child using the forest.	CO6.3. The expectation for life with children is increased.	CO6.4. Clarify when and how to implement the plans with children.

CO = Change Objective; PO = Performance Objective.

**Table 4:** Theoretical Methods, Strategies, and Practical Applications.

Determinants	Representative Change Objectives, Unmarried Mothers Will:	Theoretical Methods Using Transtheoretical Model	Strategies
**Knowledge**	Improve their knowledge of forest-healing effects; Reduce negative perception of themselves; Understand how to use the forest to improve cognition and emotions.	Consciousness-raising (obtain the facts) Self-reevaluation	Providing information: small group lectures using audiovisual materials.
**Skill**	4. Express how to use forest healing factors; 5. Be aware and express their emotion and cognition; 6. Realize self-care through forest healing activities; 7. Positively communicate with others through forest healing activities.	Dramatic relief (pay attention to feelings) Reinforcement management Stimulus control	Demonstrations, practical skill training, supervision, group activities.
**Attitude**	8. Increase the expectation that the forest can improve mental and physical health; 9. Expect a healthy life with their children and others through the forest; 10. Expect psychological comfort through the forest.	Self-liberation Social liberation Counter conditioning	Group discussion, supporting, and motivation.
**Availability and Accessibility**	11. Prepare and implement specific plans for using forests to promote mental and physical health; 12. Realize positive parenting through cooperation.	Environment reevaluation Helping relationships	Group discussion, social support.

**Table 5:** Details Regarding the Composition of Each Session.

Session	Theme	PO	CO	Contents		Method	TTM Stage
				Off-Line	On-Line		
**1**	Pleasant meetings in forests	PO1 PO2	CO1.1.	Explanation of forest healing effects and methods.		Small group lecture	Contemplation and Preparation
			CO1.2 CO2.1.a.	Exercise breathing to accept phytoncide in the forest.		Group activity demonstration	
			CO1.3	Share expectations of the forest program.		Group activity	
			CO1.4 CO2.4.	Take a walk in the nearby forest.	Making a happiness pot (growing cherry tomatoes).	Individual activity	
**2**	Forest of reflection	PO1 PO2	CO1.3. CO2.2.a.	Stretching in the forest.	Stretching with the forest sound.	Guided practice	
			CO2.1.b.	Find a tree that resembles me.	Find leaves that I like.	Individual activity	
			CO1.4. CO2.2.b. CO2.3.a. CO2.3.b.	Find a tree that resembles me and talk about my emotions.	Express myself and share my emotions using natural objects; Assignment: take a walk in the forest and take a photo of my favorite landscape.	Group activity	
**3**	Forest of care	PO1 PO3	CO1.3. CO1.4 CO3.1.a. CO3.2.a. CO3.2.b.	Five-sense walk in the forest: the feeling of touching leaves and trees, and the smell of soil; Sensory activities in the forest focusing on the visual, tactile, and olfactory senses.	Forest meditation: image meditation while sitting in a comfortable seat recalling a favorite landscape in the forest.	Guided practice	Action
				Take a foot bath after walking barefoot (safe and clean space).	Sensory activities in the forest focusing on the visual sense; Take a footbath while listening to the sound of the forest.		
**4**	Forest of achievement	PO1 PO3	CO1.4. CO3.3. CO3.4.	Gardening craft Moss terrarium, Tillandsia air plant		Individual activity	
			CO3.2.b.	Self-praise on the work completed on the gardening crafts and praise and encouragement among members.		Group activity	
**5**	Forest of comfort 1	PO1 PO4	CO1.4. CO4.1.a. CO4.2.a.	Five-sense walk in the forest: the sound of leaves swaying, the sound of birds chirping, and the natural scent in the forest-auditory sense-centered forest sensory activities.	Forest ASMR meditation: indirect sensory activities in the forest through playing a video, closing eyes, and listening to forest sounds- auditory sense-centered forest sensory activities	Guided practice	
			CO1.3.	Nanta beating in the forest: practice Nanta beating using natural objects available in the forest, such as leaves and branches.	Forest meditation: loving-kindness meditation creating a kind heart wishing happiness, safety, and comfort for all living things. Assignment: take a walk around the path in an urban forest and take a picture in the forest	Individual activity	
			CO4.1.a. CO4.2.a. CO4.3.b.	Healing tea therapy: sharing the forest experience with healing tea.		Group activity	
**6**	Forest of comfort 2	PO1 PO4	CO4.1.b. CO4.2.b.	Becoming a valuable instrument and brushing off each other’s/my dust.		Group activity	
			CO1.4. CO4.3.a.	Walk in the forest (use a mirror to see the forest and turn around to view the forest).	After a walk in an urban forest path, share the experience of taking pictures in the forest.	Individual activity	
			CO4.4.	Five-sense activities in the forest: aroma hand massage. Sensory activities in the forest centered on olfactory and tactile senses.		Group activity	
7	Forest of happiness	PO1 PO5	CO5.1.b. CO5.2.b. CO5.3.b.	Share experiences of parenting stress.		Group activity	
			CO1.3. CO5.4.	Foliage meditation (based on mindfulness meditation): understanding foliage meditation method in everyday life.		Individual activity	
			CO5.1.a.	Craft postcards with dry flowers.		Individual activity	
8	Forest of hope	PO5 PO6	CO6.3.	Check the growth of happiness pot.		Group activity	Maintenance
			CO5.1.a. CO5.2.a. CO5.3.a.	Feel the life of seeds at my toes.		Guided practice	
			CO6.1. CO6.2. CO6.4.	Write a letter to myself: my dreams and wishes.		Individual activity	

CO: change objective; PO: performance objective; TTM: transtheoretical model; ASMR: autonomous sensory meridian response.

## Data Availability

All data generated or analyzed during this study are included in this published article.
